# Targeted Molecular Dynamics Study of C-Loop Closure and Channel Gating in Nicotinic Receptors

**DOI:** 10.1371/journal.pcbi.0020134

**Published:** 2006-09-29

**Authors:** Xiaolin Cheng, Hailong Wang, Barry Grant, Steven M Sine, J. Andrew McCammon

**Affiliations:** 1 Howard Hughes Medical Institute, University of California San Diego, La Jolla, California, United States of America; 2 NSF Center for Theoretical Biophysics, Department of Chemistry and Biochemistry, University of California San Diego, La Jolla, California, United States of America; 3 Receptor Biology Laboratory, Department of Physiology and Biomedical Engineering, Mayo Clinic College of Medicine, Rochester, Minnesota, United States of America; 4 Department of Pharmacology, University of California San Diego, La Jolla, California, United States of America; Weill Medical College of Cornell University, United States of America

## Abstract

The initial coupling between ligand binding and channel gating in the human α7 nicotinic acetylcholine receptor (nAChR) has been investigated with targeted molecular dynamics (TMD) simulation. During the simulation, eight residues at the tip of the C-loop in two alternating subunits were forced to move toward a ligand-bound conformation as captured in the crystallographic structure of acetylcholine binding protein (AChBP) in complex with carbamoylcholine. Comparison of apo- and ligand-bound AChBP structures shows only minor rearrangements distal from the ligand-binding site. In contrast, comparison of apo and TMD simulation structures of the nAChR reveals significant changes toward the bottom of the ligand-binding domain. These structural rearrangements are subsequently translated to the pore domain, leading to a partly open channel within 4 ns of TMD simulation. Furthermore, we confirmed that two highly conserved residue pairs, one located near the ligand-binding pocket (Lys145 and Tyr188), and the other located toward the bottom of the ligand-binding domain (Arg206 and Glu45), are likely to play important roles in coupling agonist binding to channel gating. Overall, our simulations suggest that gating movements of the α7 receptor may involve relatively small structural changes within the ligand-binding domain, implying that the gating transition is energy-efficient and can be easily modulated by agonist binding/unbinding.

## Synopsis

Nicotinic acetylcholine receptors are ligand-gated ion channels responsible for neurotransmitter-mediated signal transduction at synapses throughout the central and peripheral nervous systems. Binding of neurotransmitter molecules to subunit interfaces in the N-terminal extracellular domain induces structural rearrangements of the membrane-spanning domain permitting the influx of cations. A full understanding of how the conformational changes propagate from the ligand-binding site to the pore domain is of great interest to biologists, yet remains to be established. Using a special simulation technique known as targeted molecular dynamics, Cheng and colleagues probed the early stages of ligand-induced conformational rearrangements that may lead to channel opening. During the simulation, Cheng et al. observed a sequence of conformational changes that stem from the ligand-binding site to the transmembrane domain resulting in a wider channel. From these results, they suggest that gating movements may entail only small structural changes in the ligand-binding domain, implying that channel gating is energy-efficient and can readily be modulated by the binding/unbinding of agonist molecules.

## Introduction

Nicotinic acetylcholine receptors (nAChRs) belong to a family of ligand-gated ion channels that bind neurotransmitter molecules [1,2], and mediate fast signal transmission at synapses throughout the central and peripheral nervous systems. They are pentamers formed from either a single type of subunit or from different types of homologous subunits. Each subunit is composed of a conserved N-terminal ligand-binding domain followed by three transmembrane helices (denoted M1 to M3), a variable cytoplasmic loop, a fourth transmembrane domain (M4), and a short C-terminal extracellular region. There are two main subtypes of nAChRs present in the nervous system: the neuronal type receptors, found in the central nervous system and on all autonomic ganglia, and the neuromuscular type receptors, present in the neuromuscular junctions of somatic muscles. Recently, neuronal nAChRs such as α4β2 and α7 have emerged as attractive therapeutic targets for the treatment of pain, cognitive impairment, neurodegenerative disease, schizophrenia, epilepsy, anxiety, and depression because of their modulatory influence in the central nervous system [3–6].

Upon agonist binding at subunit interfaces, the ligand-binding domain of the nAChR undergoes conformational rearrangements that propagate to the membrane-spanning domain and cause opening of the ion channel. Details of agonist-induced conformational changes within the ligand-binding domain have recently been obtained from crystallographic structures of related acetylcholine binding proteins (AChBPs) [7–11]. The comparative analysis of an apo AChBP structure with several agonist complexed structures indicates that large conformational changes are localized to loops C and F [11]. The C-loop behaves as a ligand-triggered lid, which moves by ~4 Å, effectively closing the ligand-binding pocket. The structure of agonist-bound AChBP also revealed substantial reorientation of several residues in the binding site associated with C-loop rearrangement. Nevertheless, as only minimal structural changes have been seen near the extracellular-membrane linkage regions, the structural mechanism that couples small changes at the ligand-binding domain to the channel gate remains elusive. In addition, despite their obvious homology the pharmacological differences between nAChRs and AChBPs may indicate that the structural rearrangements at the bottom of the ligand-binding domain differ in the two proteins.

Additional information on the dynamics of the ligand-binding domain has been provided by chemical probe and mutagensis methods [12,13]. Recently, by determining energetic couplings between pairs of residues through single-channel current recordings, Sine et al. showed that the initial coupling of binding to gating was mediated by the conserved residue triad αLys145, αTyr190, and αAsp200 in the muscle nicotinic receptor [14]. The same investigators further demonstrated that a pair of conserved residues, Arg209 and Glu45, are electrostatically coupled in linking agonist binding to channel gating [15]. Significant steps have also been made toward uncovering the transitions that occur during gating within the transmembrane domain. Employing unnatural amino acid mutagenesis, Lummis et al. showed that a switch in proline from the *trans* to the *cis* conformation might open the pore of a neurotransmitter-gated ion channel [16]. Site-directed mutagenesis of the pore-lining M2 domain, combined with kinetic analysis, suggested a simple pore-widening mechanism for gating [17], rather than the previously proposed mechanism involving rotation of the M2 helices [17,18].

**Figure 1:**
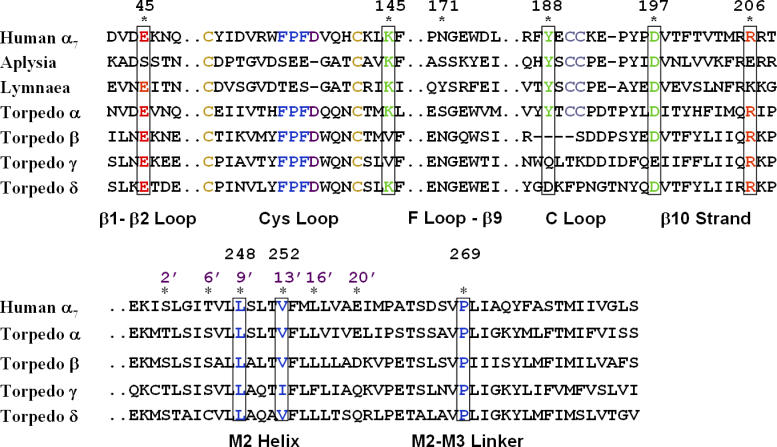
Amino Acid Sequence of the Human α7 Nicotinic Receptor Aligned with AChBP and the *Torpedo* Receptor Sequence numbering corresponds to the human α7 receptor. Positions colored red and green are highly conserved, and have been implicated in the channel gating mechanism.

In this study, targeted molecular dynamics (TMD) simulation was performed on the human α7 receptor to investigate the initial coupling of C-loop closure to channel gating, and to determine how residues Arg206 (equivalent to Arg209 in the muscle receptor; see the sequence alignment in Figure 1) and Glu45 could link binding to gating. For comparison, a 10-ns standard equilibrium molecular dynamics (MD) simulation of the same system, referred to as the control simulation below, was also conducted. MD simulations have been increasingly employed to provide insight into atomic detail as well as dynamical aspects of structural mechanisms, which are often difficult to achieve by experiment alone [19]. Since the availability of the Cryo-EM structure of *Torpedo* nAChR, a number of MD studies have been carried out on the nicotinic receptors. Henchman et al. studied the dynamics of the ligand-binding domain of the human α7 nicotinic receptor, and observed asymmetrical motions in the presence of different ligands [20]. MD simulations of the transmembrane domain investigated the dynamics of the M2 helices [21–23], the importance of the hydrophobic gates [24,25], and the lipid–M4 interactions [26]. More recently, a simulation on the human α7 nAChR with both the ligand-binding and transmembrane domains revealed that the protein undergoes a twist-to-close motion that correlates movements of the C-loop with the rotation and inward movement of two nonadjacent subunits [27]. Despite these advances, a major limitation for the application of MD simulation to the study of nAChRs is that the time scale for the gating transition (μs–ms) is beyond the range of current computational tractability. Therefore, in this study, TMD simulation was used to accelerate the conformational transition from the inactive to the active structure [28]. The current TMD simulation was initiated from a homology model of the human α7 receptor based on the Cryo-EM structure of nicotinic receptor from Torpedo marmorata [29]. During the simulation, the C-loops of two alternating subunits (corresponding to the two α subunits in the *Torpedo* receptor) were forced to move toward the ligand-bound conformation (as represented by the crystallographic structure of agonist-bound AChBP [8]) through restraining forces applied to eight residues at the tip of the C-loop (see Materials and Methods). An important assumption is that the C-loop regions of the nAChR and AChBP undergo similar structural rearrangements after acetylcholine binding. Despite the minimal rearrangements distal from the ligand-binding site in ligand-bound AChBP, we observe large conformational changes toward the bottom of the ligand-binding domain in the nAChR upon forced C-loop closure. These structural changes are translated to the pore domain, leading to a partly open channel within 4 ns of TMD simulation.

## Results/Discussion

### Conformation of the C-Loop

**Figure 2:**
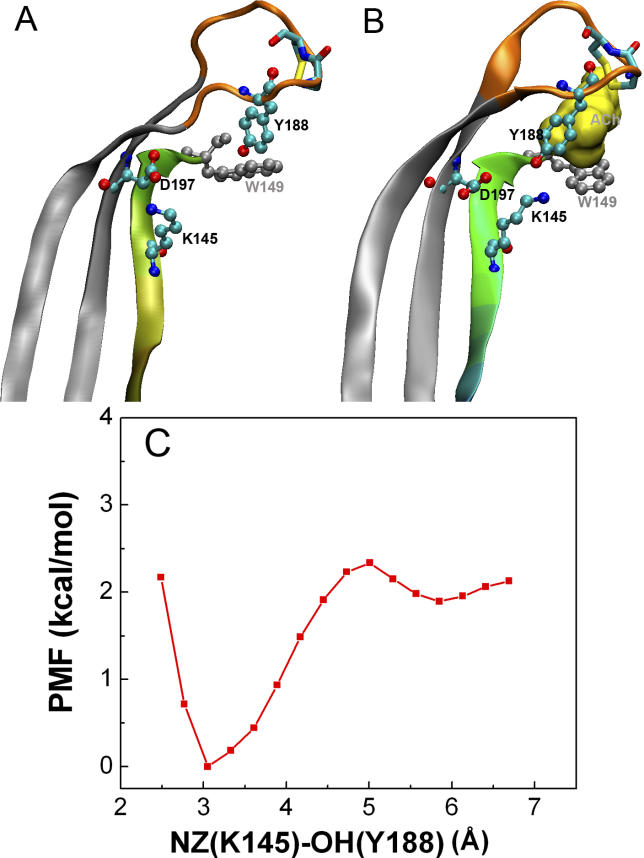
Structural Comparison of apo and ACh-Bound Conformations of the Ligand Binding Domain of Human α7 Receptor The triad of conserved residues is shown in stick representation. The tip of C-loop, to which the targeted force is applied (residues Arg186-Glu193), is colored orange. The apo (A) conformation is the homology model based on the *Torpedo* α subunit, while the ACh-bound (B) conformation is modeled based on the crystal structure of AChBP with bound carbamoylcholine [8]; (C) the PMF for the interaction between Lys145 and Tyr188 (see text).

The ligand-bound conformation of the C-loop, as captured in the crystallographic structure of AChBP in complex with carbamoylcholine [8], was used as the target structure in our TMD simulation (Figure 2A and 2B). The root-mean square deviation (RMSD) between the C-loop (based on C^α^ atoms of residues 183–200) in the initial and target structures was ~4.3 Å. This RMSD was gradually reduced, via the application of energy restraints, resulting in an “open” to “closed” C-loop transition, which corresponds to a conformational transition from the inactive to a putative active state. After 2 ns of TMD simulation, the C-loop folded over the ligand-binding site, with an RMSD relative to the target structure of ~0.4 Å. The C-loop remained in the closed conformation for the subsequent 2-ns relaxation period. There was little lag or structural distortion during the transition, indicating that a relatively low energetic barrier separates the two C-loop conformations.

C-loop closure resulted in a small-scale structural reorganization of several residues in the vicinity of the ligand-binding pocket. These rearrangements resulted in the formation of a hydrogen bond between Tyr188 in the C-loop and Lys145 in β strand 7. During the TMD simulation, the distance between NZ(Lys145) and OH(Tyr188) decreased from 5.7 Å to 3.1 Å. The final distance of 3.1 Å is similar to that observed in the crystallographic structure of ligand-bound AChBP [8]. To investigate the strength of the Lys145-Tyr188 hydrogen bond and the possibility that its formation may contribute to C-loop closure, the effective free energy (potential of mean force (PMF)) was calculated using umbrella sampling of the NZ-OH distance. The agonist-bound conformation was stabilized by ~1.9 kcal·mol^−1^ when forming a hydrogen bond at distance of 3.0 Å (Figure 2C), comparable to the experimentally measured coupling energy between Lys145 and Tyr188 of 2.6 kcal·mol^−1^ [14]. The strength of the hydrogen bond was somewhat weaker than expected, probably due to the flexibility of the C-loop and the intimate interactions of both residues with water molecules when not interacting. The weak interaction of Lys145 with Tyr188 may help explain why the mutation of either or both residues reduces channel gating efficiency but does not abolish it completely [14]. In addition, there are probably other residues or motions, such as the rotation of the inner core of the β strands, that contribute to coupling ligand-binding to the channel pore.

### Initial Response to C-Loop Closure

**Figure 3:**
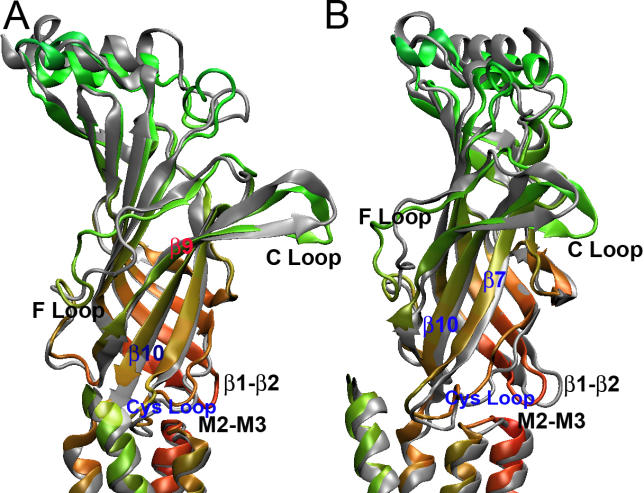
Structural Changes of β10, Cys loop, and β1–β2 loop during the TMD Simulation Comparison of the initial structure (silver) and a snapshot from the last 50 ps of the TMD simulation (green), (A) front view; (B) side view rotated 120° with respect to view (A).

Immediately following the inward motion of the C-loop, strands β9 and β10 were observed to undergo a sliding motion, which shifted the lower portion of strand β10 in the vicinity of Arg206. This rearrangement resulted in an average upward displacement of Arg206 ~1.5 Å from the membrane surface, as well as an outward displacement of ~1.0 Å from the channel axis (Figure 3). Although the amplitude of the movement was small, it turned out to be sufficient to initiate the subsequent conformational changes that resulted in pore widening.

**Figure 4:**
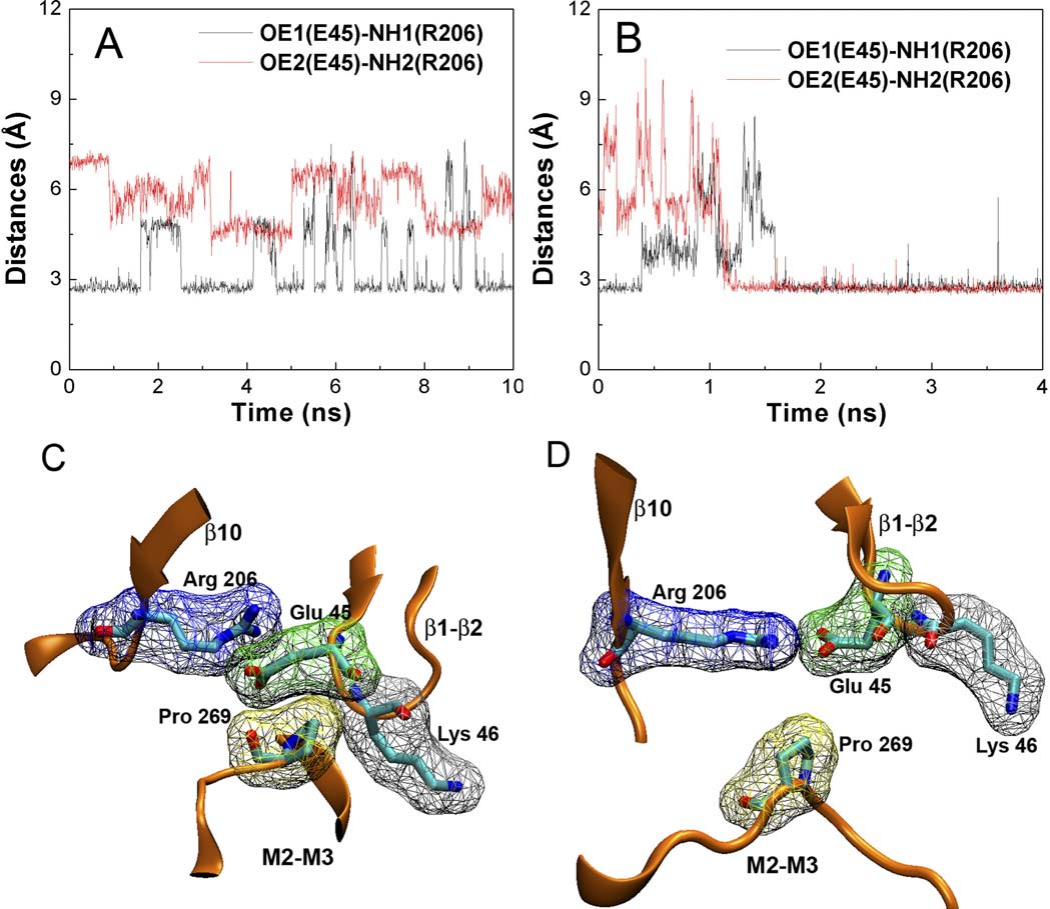
The Hydrogen Bond Distances between Arg206 and Glu45 (A) During the 10-ns control simulation. (B) During the 4-ns TMD simulation. Detailed view of residues Arg206, Glu45, Lys46, and Pro269 in the starting structure (C) and in the final conformation from the TMD simulation (D).

**Figure 5:**
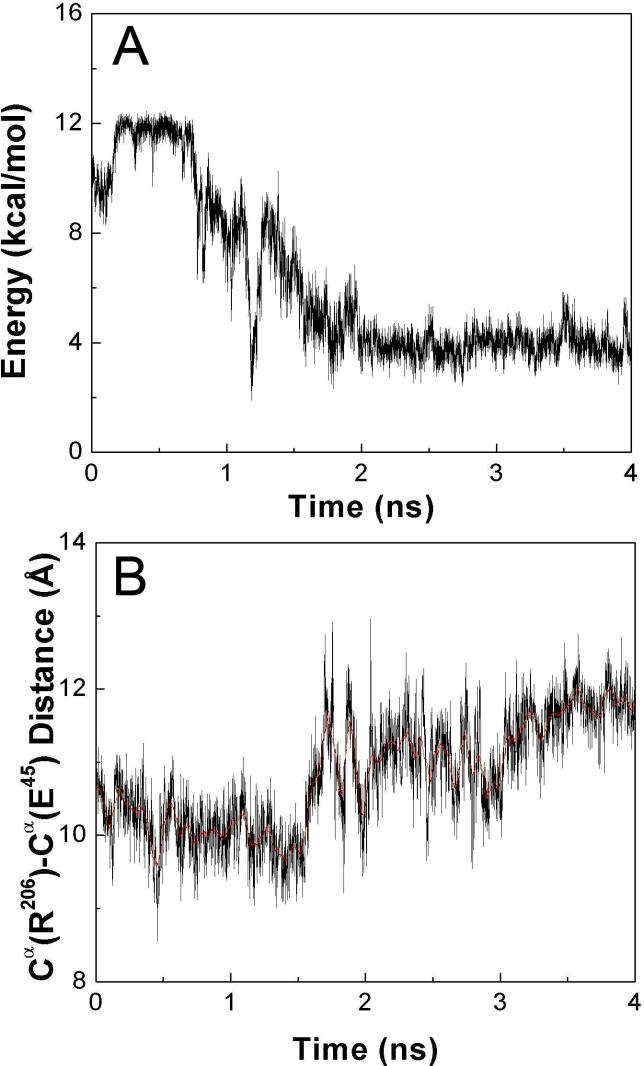
Energetic and Structural Changes of Several Key Residues at the Membrane Interface during the TMD Simulation (A) Estimation of the nonbonded interaction energies between Glu45, Lys46, and Pro269 during the TMD simulation. (B) C^α^(R206) –C^α^(E45) distance as a function of time during the TMD simulation.

The tip of the β1–β2 linker underwent an inward motion toward the pore center following the lower part of the β10 strand. Initially, this motion was sterically obstructed by the top of the M2–M3 linker; notably by Pro269, which stayed in close contact with Glu45 and Lys46 from the β1–β2 linker (Figure 4). Removal of the steric obstruction between these residues permitted an ~10° rotation of the M2–M3 linker during the TMD simulation. This rotation is reflected in the change of the nonbonded interaction energies displayed in Figure 5A. Once β1–β2 passed by the M2–M3 linker, the contacts between M2–M3, β1–β2, and Cys loops became less extensive. At the same time, a more stable hydrogen bond was established between Arg206 and Glu45 (Figure 4B and 4D).

To ascertain that the movements of the β10 strand, M2–M3, and β1–β2 loops were indeed triggered by the forced rearrangement of the C-loop, we examined the motions of these regions in a 10-ns control MD simulation during which force was not applied. During the control simulation, the C-loop moved farther away from the binding pocket, coupled with a slight bending motion of the β10–M1 hinge at the N-terminus of M1. Residues 205–207 (located at the hinge region) shifted toward the pore center, moving in an opposite direction compared with the same residues in the TMD simulation. Furthermore, the backbone of residues 45 and 46 on β1–β2 stayed relatively invariant. In contrast to the rotational movements observed in the TMD simulation, the M2–M3 linker underwent a downward curling motion toward the membrane surface. This motion was dynamically uncorrelated with the nearby β1–β2 region.

**Figure 6:**
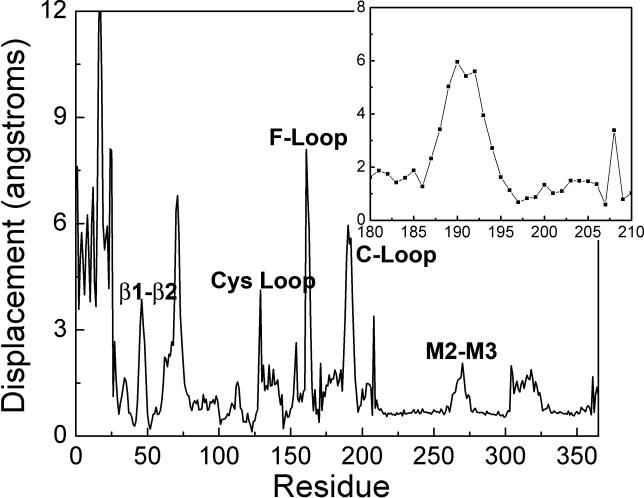
The RMS Fluctuations of C^α^ Atoms during the TMD Simulation For clarity, only results for subunit A are shown. The inset shows the displacements of the β9 and β10 strands. Asp197 remains relatively stationary during the enforced inward motion of the C-loop, as indicated by its low displacement value.

The C^α^ RMS fluctuations during the TMD simulation are shown in Figure 6. Most regions display relatively small fluctuations. Notable exceptions are the C-loop (residues 186–193), F-loop (residues 160–170, β8–β9), and Cys loop (residues 128–142). Asp197 remained relatively invariant; it maintained its initial hydrogen bond with Lys145 while forming a second hydrogen bond with the incoming Tyr188. Considerable fluctuations were evident for the middle part of the F-loop, consistent with the appearance of disorder in the Cryo-EM structure [29]. Our TMD simulation also showed the F-loop rotating slightly outward from its initial position. A striking consequence of this rotation was that Asn171 became more solvent-exposed. Initially Asn171 was engaged in extensive electrostatic interactions with residues Lys46, Asn47, and Asp131 from neighboring subunits. The removal of such contacts is consistent with recent hydrophobic photolabeling experiments that suggested the F-loop changes its local environment during channel gating [12].

The conserved Cys-loop is indispensable for coupling agonist binding to channel gating [30,31]. In our TMD simulation, the entire loop tended to move closer to β10–M1. However, the highly conserved FPFD motif (residues 135–138), which extends down to the tops of the four transmembrane helices, restricted the movement of the Cys-loop, causing a slight twisting at the loop center. One of the residues next to the FPFD motif, Trp134, was found to depart from the plane of the membrane surface, also in agreement with photolabeling experiments [12]. Although direct evidence is lacking from the current TMD simulation that the movements of the Cys-loop and the M2–M3 linker are dynamically coupled, the close contact maintained between Phe135 (located in the Cys loop) and Ile271 (located in the M2–M3 linker) during simulation, suggests that the Cys-loop may act as a pivot point for the movement of M2–M3 linker.

### Interaction between Arg206 and Glu45

**Figure 7:**
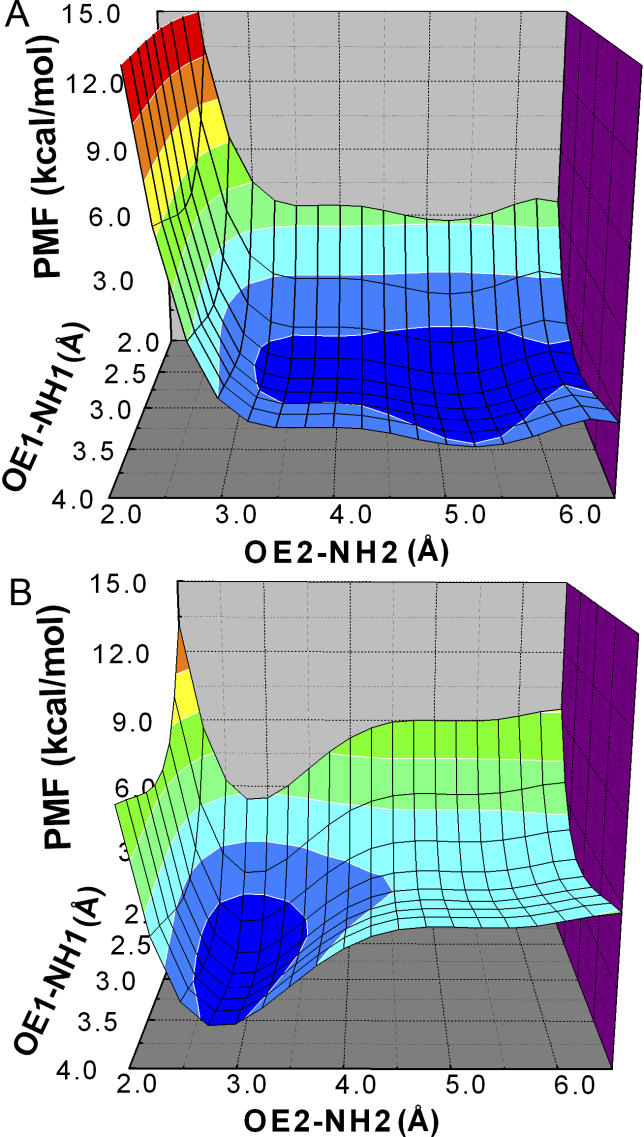
The PMFs for the Interaction between Glu45 and Arg206 (A) In the starting structure. (B) In the final conformer from the TMD simulation. PMF energy is shown on a continuous color scale from 0 (blue) to ≥ 10 kcal/mol (red).

It has previously been proposed that a coupling between Arg206 and Glu45 plays an important role in coupling agonist binding to channel gating [15]. In the current TMD simulation, this interaction seemed essential to mediate the concerted motion of the β10–M1 (Arg206) and β1–β2 regions (Glu45). This interaction appeared to cause the lower part of the β10 strand to draw the β1–β2 loop away from its original location. When the receptor was in the initial resting conformation, one hydrogen bond was formed between Arg206 and Glu45. This hydrogen bond broke spontaneously over the course of the 10-ns control simulation (Figure 4A and 4C). However, after ~1.5 ns of TMD simulation, the C^α^–C^α^ distance increased by ~1 Å (Figure 5B). At this time, two more favorable hydrogen bonds formed between Arg206 and Glu45 (Figure 4B and 4D). Taken together with mutagenesis experiments demonstrating a salt bridge between Arg206 and Glu45 [15], the present results suggest that a stronger salt bridge is associated with the active relative to the inactive conformation. To determine the interaction strength between Arg206 and Glu45, we calculated the effective free energy using NH1-OE1 and NH2-OE2 distances as two reaction coordinates. Two backbone conformations were investigated, one corresponding to the initial resting structure (Figure 4C) and a second corresponding to the final conformer from the TMD simulation (Figure 4D). It was found that with the initial backbone conformation, only one hydrogen bond was possible with a favorable interaction of 2.3 kcal·mol^−1^ (Figure 7A). Hydrogen bonding was 3.7 kcal·mol^−1^ more stable as Arg206 and Glu45 became separated by ~1 Å (Figure 7B), consistent with the experimental coupling energy of ~3.1 kcal·mol^−1^ [15]. It should be noted that the experimental free energy associated with Arg206-Glu45 coupling was measured by applying mutant cycle analysis to the channel gating step. This coupling energy originated from the set of mutations Arg to Gln and Glu to Arg, and would likely have been even greater if a complete mutant cycle for the Arg to Glu and Glu to Arg set could have been generated [15].

Previously, it has not been possible to infer structural details of the receptor gating mechanism because no significant conformational changes beyond C-loop displacement are evident in the AChBP crystal structures [11,20,32]. In the current TMD simulation, the movements of Arg206 and Glu45 were clearly coupled. It is likely that mutation of either residue would disrupt the propagation of a conformational change from the binding to the pore domain. In the crystal structure of AChBP [8,11], Lys206 (equivalent to Arg206 in nAChRs; see sequence alignment in Figure 1) clearly points away from its partner, whereas the orientation of Glu45 (or Ser45 in *Aplysia* AChBP) is similar to that in nAChRs. Moreover, since Lys206 is located at the C-terminus, this region of the structure may be of insufficient accuracy to depict the differences in the presence or absence of agonist.

### Dynamics of the Transmembrane Pore

**Figure 8:**
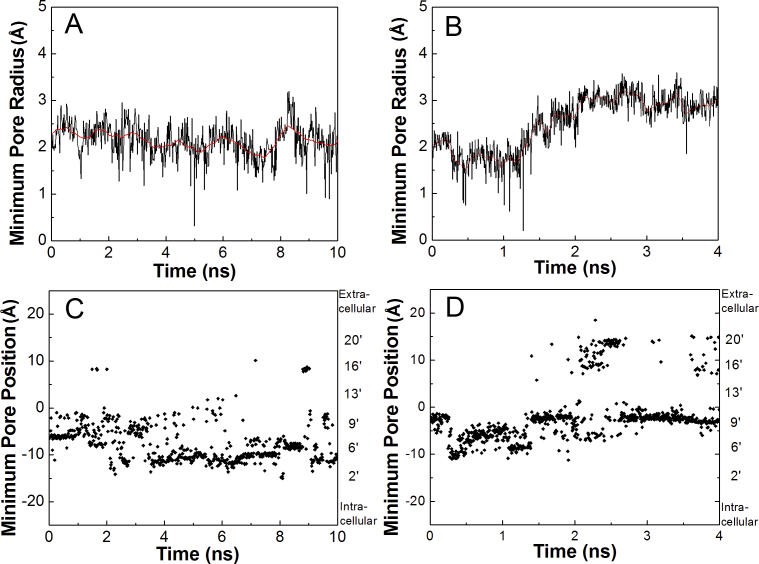
Minimum Pore Radius and *z*-Axis Position of the Minimum Pore Radius as a Function of Time (A,C) During the control simulation. (B,D) During the TMD simulation. The center of the pore domain is set to zero, with positive *z* values toward the extracellular end. The conventional numbering for M2 residues is depicted on the right axis.

Ion conduction occurs upon conformational rearrangements of the pore-lining helices that open the channel. In the current TMD simulation, motion of the pore domain gradually increased the radius of the central vestibule (quantified in Figure 8). This vestibule widening lagged behind the closure of the C-loop of the ligand-binding domain, implying the existence of a sizeable energetic barrier (>1.2 kcal·mol^−1^). This observation is consistent with the proposal that an intermediate state may exist between the ligand-binding domain transition and channel gating [33]. In comparison, pore size remained relatively invariant throughout the control simulation (Figure 8A). This is consistent with previous equilibrium simulations where only minor pore radius fluctuations were noted [25]. Thus, the pore widening observed in the current TMD simulation may be a direct consequence of C-loop closure.

The steric interaction of the β1–β2 loop with the M2–M3 linker appeared to be a major factor for initiation of pore widening. However, as the motion of the M2–M3 linker and the β1–β2 loop occurred almost simultaneously in the TMD simulation, it is also possible that the displacement of the Arg206–Glu45 pair removed steric interactions that would have restricted movement of the pore-lining helices. As illustrated in Figure 4D, the β10–M1 and β1–β2 linkage disengaged from the M2–M3 linker after the TMD simulation. This disengagement might free the M2–M3 linker to rotate and allow the M2 helices to move. Thus the β10–M1 and β1–β2 linkage in the resting state might simply block an intrinsic tendency of the channel to open. To help clarify this issue, we conducted a complementary simulation on the transmembrane domain of the α7 receptor where the steric obstruction (i.e., the Cys loop, the β10, and the β1–β2 linkage) was removed by deleting the extracellular domain. After 10 ns of standard equilibrium MD simulation, no pore size increase was evident (unpublished data). Thus, pore opening in our TMD simulation is likely driven by rotation of the β1–β2 loop. Nevertheless, the effect of removing steric hindrance from the bottom of the ligand-binding domain cannot be completely discounted, due to the limited time scale of the complementary simulation.

The time evolution of the minimum pore size and the corresponding *z*-axis position during the TMD simulation is depicted in Figure 8B and 8D. Toward the beginning of the simulation (from 0.5–1.2 ns), the minimum pore size decreased slightly (from 2.0 Å to 1.7 Å), indicating a slight narrowing of the pore. Closer inspection revealed that this was due to side-chain movements at the intracellular end of the pore. After this initial lag (~1.2 ns), the minimum pore size started to increase (assuming a value of 3.0 ± 0.3 Å). Interestingly, the minimum pore position shifted during the TMD simulation. At the beginning, the minimum pore radius localized near the middle of the pore (positions 9′ and 13′). As the simulation progressed and the minimum pore radius increased, the narrowest part of the pore fluctuated between positions 9′ and 13′ and between positions 16′and 20′. Notably, the pore around position 16′ did not narrow, but rather its size did not increase to the same extent as in the vicinity of positions 9′ and 13′. This observation is consistent with a recent Cryo-EM structure of *Torpedo* nAChR, indicating that the minimum pore position localized to between positions 9′ and 13′, and that the channel may be gated by the rotation of the corresponding residues [18]. In addition, photolabeling and substituted-cysteine accessibility experiments have provided compelling evidence that some pore lining residues change their solvent accessibility during channel gating [34–37].

It was previously suggested that the M2 helices undergo a rigid-body rotation during gating [18]. However, the precise motions of the helices are still largely unknown. We have used two different parameters to quantify the overall motion of the M2 helices during the TMD simulation. Namely, the orientation angle of a helix relative to the channel axis, and the rotation of a helix around the channel axis (see Materials and Methods). We observed both tilting and rotational motions during the TMD simulation (Table 1). Given that external force was applied to subunits A and D, these subunits were expected to display the greatest movement. This seems to be true for rotational movement. Both A and D subunits rotated clockwise by ~7°. However, subunit C also rotated by ~4°. The extent of tilting for each M2 helix did not appear to depend on the external forces; all M2 helices became slightly more tilted (3°–6°) in the putative open state.

**Table 1:**
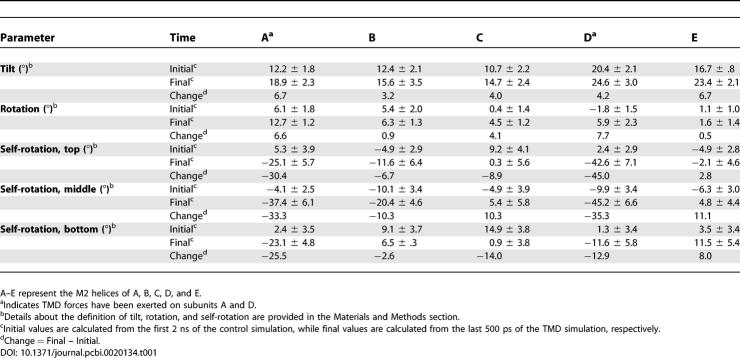
Parameters That Describe the Motion of M2 Helices during TMD Simulation

Parameters describing simple rigid-body rotation and tilting of the helix may not be sufficient to describe more complex motions in the pore domain [17,21,38,39]. Thus a self-rotation angle parameter was developed to monitor the movements of individual pore-lining residues (Table 1). This analysis appeared to be more informative. Although equivalent residues (at the same M2 position) in different subunits rotated asymmetrically during the TMD simulation, a general trend was apparent with more negative rotation angles for residues in the A and D subunits. That is, those residues that face the channel lumen in subunits A and D rotated clockwise by ~30°, which seems sufficient to remove the obstructing hydrophobic side chains. Notably, all three residues (bottom: 2′, middle: 13′, and top: 20′) in the same M2 domain rotated in the same direction, albeit with different magnitudes. Further analysis revealed that the side chains of Val252 (position 13′) possibly changed their accessibility to the lumen while the side chains of Gly241 (position 2′) and Glu259 (position 20′) remained accessible to the lumen. Overall, the self-rotation analysis strongly implies that a rotational motion is involved in pore widening while disfavoring the possibility of a pure pore expanding or tilting mechanism. In addition, the decreased magnitude of the helix rotation and tilt parameters compared with the self-rotation measurement suggests that the motion cannot be described as a simple rigid-body rotation, but that it also involves the tilting, bending, or both of the M2 helical structure.

### Hydration Properties of the “Open” Transmembrane Pore

**Figure 9:**
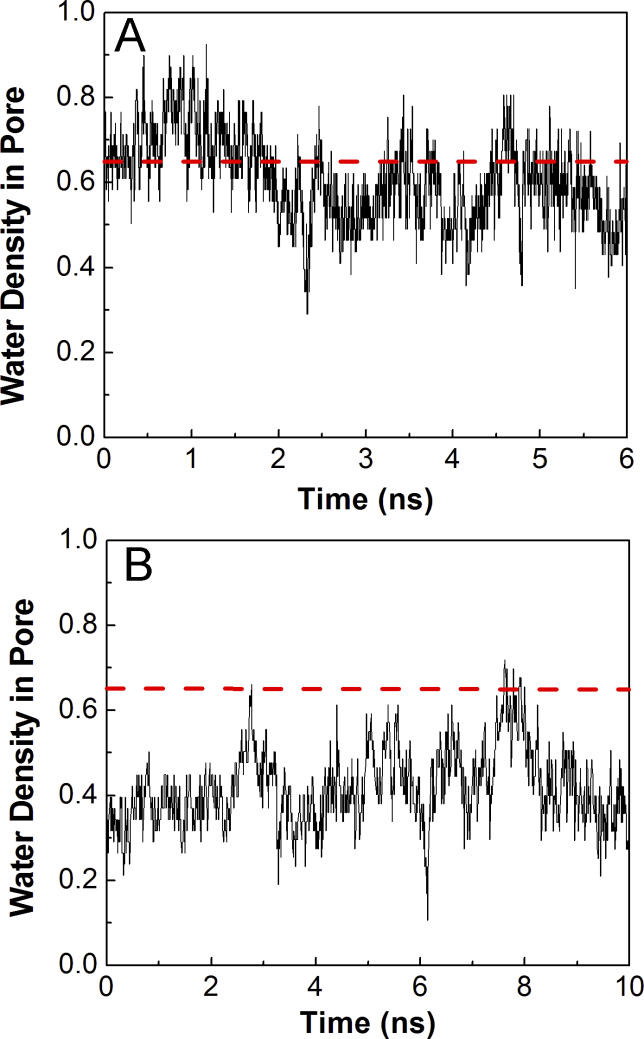
Water Density in the Central 5-Å Portion of the Pore Normalized by the Bulk Water Density (A) During the simulations of the final TMD conformer. (B) During the simulations of the control simulation. The red dashed lines indicate the water density at 0.65 of the bulk density.

Given a targeted structure with a widened pore size, it was of interest to examine whether this conformation was capable of ion conduction. Rather than performing an ion-pulling simulation [40,41], we conducted 6 ns of MD simulation starting from the final conformer of the TMD simulation. Presumably, an open pore must be filled with water molecules in order to conduct ions, as the pore lumen is mainly lined with hydrophobic residues [25,42]; otherwise an isolated sodium ion entering an empty hydrophobic pore would experience a significant energetic barrier [43]. In Figure 9, the time evolution of the water density in the pore during the simulation of the TMD final conformer is depicted along with that from the control simulation. Earlier simulations suggested that the water density in the pore can be used as an indicator of the conductance state of the channel [25,42]. Using 0.65 of the bulk density as a threshold for water permeation as in Sansom et al. [42] revealed that the channel remained nonconductive for the majority of the control simulation. But in the simulation of the TMD final conformer, due to the increase of the pore size, the water density in the pore increased considerably as compared with that in the control simulation. Although the channel appeared to reach the conducting state at the start of the TMD simulation, the water density dropped below 0.65 of the bulk density most of the time after ~2 ns of simulation. This indicates that the TMD final conformer may still not be representative of a completely open state even though the pore had widened by ~1 Å.

**Figure 10:**
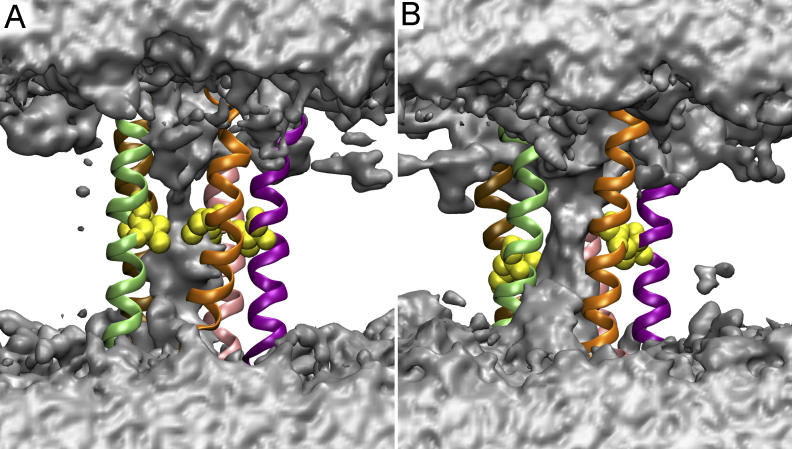
The Iso-Surfaces of Time-Averaged Water Density (A) During the control simulation. (B) During the the simulation commencing from the final TMD conformer. The surface corresponds to the iso-density contour ~0.4 of the bulk water density. Five M2 helices are shown in ribbons. Residue Val252 is highlighted as yellow spheres and displaced sideways in the final TMD conformer, contributing to the higher water density observed for this simulation.

The iso-surfaces of time-averaged water density from both the control simulation and the simulation of the TMD final conformer are shown in Figure 10. Consistent with the density profiles in Figure 9, the simulation with the TMD final conformer resulted in a higher water occupancy than in the control simulation. Furthermore, the water density volume showed a clear constriction for water passage near Val252 (position 13′) in both simulations. However, closer inspection of the trajectories revealed that the side chain orientations as well as the backbone conformations of Val252 were different in the two simulations (Figure 10). The hydration simulations, therefore, suggest that the initial constriction at Leu248 and Val252 has been partially removed, but that still further conformational rearrangements of the membrane-spanning segment may be required for ion conduction. Due to time scale limitations, such structural changes were not observed within the 4 ns of our TMD simulation.

The difference in hydration for the two systems may be tentative. Previous simulations on a simplified nanopore system demonstrated that ionic charge imbalance and the potential across the membrane can induce water permeation of a hydrophobic pore [43]. From this perspective, further computational studies employing more realistic conditions (i.e., with a suitable membrane potential) may be required to identify different hydration states of the channel.

### Conclusions and Implications

**Figure 11:**
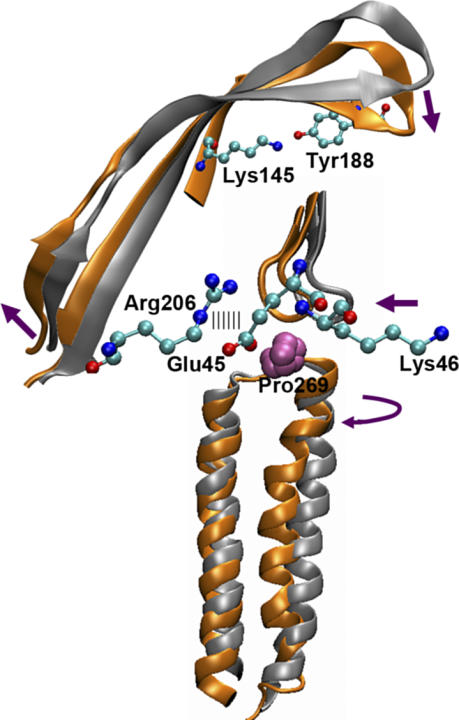
Sequence of Conformational Changes in the Ligand-Binding and Transmembrane Domains of the Human α7 Receptor The resting and activated conformations are shown in silver and orange, respectively. Glu45, Lys46, Lys145, Tyr188, and Arg206 are shown in ball-and-stick representation. Pro269 is shown in sphere representation.

The current TMD simulation of forced C-loop closure highlights a sequence of structural changes that resulted in a wider pore, and may be of relevance for understanding how agonist binding is coupled to channel gating. As illustrated in Figure 11, C-loop closure induced an upward and outward motion of the lower portion of strand β10, which was subsequently conveyed to the M2–M3 linker through the strong coupling between Arg206 and Glu45. The results of our simulation highlight two residue pairs, one located near the ligand-binding pocket (residues: Lys145 and Tyr188), the other located toward the bottom of ligand-binding domain (residues: Arg206 and Glu45), as potentially important for coupling agonist binding to gating. Within our 4 ns TMD simulation, the pore size increased from ~1.9 Å to ~3.0 Å, with the initial hydrophobic obstruction largely removed at Leu248 and Val252 (positions 9′ and 13′). The M2 domains from subunits A and D were observed to undergo a ~7° clockwise rotation, probably owing to the torque exerted on their extracellular ends by the β1–β2 loops. Overall, the observed motions, such as agonist-induced collapse of the C-loop over the binding site, the rotation of M2, and conformational rearrangements transmitted through β10 to the transmembrane domain, are consistent with previous computational studies of the receptor [27,44,45]. Together, these studies strongly suggest that rotation rather than a simple tilting or kinking of M2 might mediate the gating mechanism. Finally, our calculations also suggest that gating movements result from only small structural rearrangements in the ligand-binding domain, implying that the transition between the closed and open states is very energy-efficient, and can easily be modulated by the binding and unbinding of agonist molecules.

## Materials and Methods

### Homology modeling

Two homology models of the human α7 receptor were constructed with Modeller version 8.0 [46,47]. The first model, which was used as the starting structure in both the TMD and control simulations, was based on the recent 4.0 Å resolution Cryo-EM structure of *Torpedo* nAChR [29]. The second model, which was intended as the target structure in the TMD simulation, was constructed by combining the X-ray structure of AChBP [8] from Lymnaea stagnalis and the Cryo-EM structure of the *Torpedo* nAChR. Details of the modeling procedure employed for construction of the first model have been described previously [45]. Briefly, the modeled structure contained 1,835 residues comprising the ligand-binding and transmembrane domains as well as part of the cytoplasmic vestibule domain between M3 and M4. Five-fold symmetry was not imposed when modeling the pentamer structure. In the first model, the C-loops in two alternating subunits had the open conformation, according to the conformations of the two α subunits of the *Torpedo* structure, while the remaining three subunits were in the more contracted C-loop conformation based on the conformations of the βγδ subunits.

Construction of the second model (target structure) involved two steps. First, a homology model of the human α7 ligand-binding domain was built from the AChBP structure with carbamylcholine bound [8]. Then the full receptor was modeled by simultaneously using the newly built ligand-binding domain and the *Torpedo* receptor structures [29] as templates. Since the ligand-binding domain template already had the same sequence as that of the target, the corresponding coordinates were directly transferred to the final model. Accordingly, the C-loops in all five subunits were in the closed conformation as seen in the 1UV6 crystallographic structure. As a result of this two-step procedure, the two templates were joined by implicitly satisfying the geometry constraints, thereby avoiding any errors from manually overlaying the two domains. All the obtained models were evaluated with PROCHECK [48] and Prosa 2003 [49]. The resulting model with the lowest Modeller target function compared favorably with the template 2BG9 structure in terms of both PROCHECK scores (93%) and PROSA energies (−0.6); the quality of the ligand-binding domain was slightly better than that of the transmembrane domain.

### MD simulations

The control MD simulations were performed with the nAChR models embedded in a fully hydrated, 120 Å × 120 Å palmitoyl-2-oleoyl-sn-glycerol-phosphatidylcholine (POPC) bilayer. This resulted in a total of ~290 POPC molecules and ~60,600 TIP3P water molecules. Charge neutralization was accomplished with the addition of 86 Na^+^ and 26 Cl^−^ ions, resulting in a 0.1-M solution. The solvated system then underwent four equilibration steps: (i) 2,000 steps of minimization with a fixed protein backbone, (ii) five cycles of a 500-step minimization with decreasing positional restraints on the protein C^α^ atoms, (iii) gradual temperature increase from 50 K to 310 K in 10,000 steps of constant-volume MD simulation with harmonic restraints (with a force constant of 3 kcal·mol^−1^·Å^−2^) on the protein C^α^ atoms, and (iv) 2-ns extensive equilibration with decreasing positional restraints on the C^α^ atoms.

MD simulations were also performed on two truncated transmembrane systems. The first corresponded to the transmembrane domain abstracted from the first homology model just described. Simulation on this system was intended to clarify the possible steric effect of β10 and the β1–β2 linker on pore opening. The second system was composed of the transmembrane domain abstracted from the final conformer of the TMD simulation. This system represents a partly “open” channel. Both simulations were performed with the proteins embedded in a fully hydrated, 120 Å × 120 Å POPC bilayer at 0.1 M ionic concentration. Nonrestrained production runs followed the aforementioned equilibration procedure.

All MD simulations were performed in the constant surface area ensemble with the NAMD2 program [50] and the CHARMM27 force field [51]. A short-range cutoff of 9 Å was used for nonbonded interactions, and long-range electrostatic interactions were treated with the Particle Mesh Ewald method [52]. Langevin dynamics and a Langevin piston were used to maintain the temperature at 310K and a pressure of 1 atm. All of the MD runs were conducted on DataStar, an IBM tera-scale machine at the San Diego Supercomputer Center.

### TMD Simulations

The TMD simulation included an additional energy term based on the RMSD of the C-loop residues during the simulation relative to a prescribed target structure. The energy term had the form: *V* = ½ ∗ *k* ∗ (*RMSD*(t)−*RMSD*_0_(t))^2^, where the force constant, *k,* was 20 kcal·mol^−1^·Å^−2^. *RMSD*(*t*) was the RMSD of the simulation structure at time *t* relative to the prescribed target structure, and *RMSD*_0_(t) was the prescribed target RMSD value at time *t*. As described above, the target conformation, toward which the C-loop was forced to move, was essentially a homology model based on the Cryo-EM structure of the *Torpedo* receptor, but with all the C-loops replaced with those in the closed conformation as seen in agonist-bound AChBP. During the simulation, the TMD forces were applied to the backbone atoms of the C-loop residues (Arg186 to Glu193). The value of *RMSD*_0_(t) was linearly decreased from 4.3 Å to 0 Å within the first 2 ns and then kept at 0 Å during the rest of the 2-ns TMD simulation. To prevent rotation of the entire molecule, the center of mass and orientation of the protein were fixed.

### PMF calculation

Taking the NZ–OH distance as the reaction coordinate, the PMF for the Lys145 and Tyr188 interaction was calculated using an umbrella sampling method [53]. The simulation was performed on a dimer of the ligand-binding domain, immersed in a 50 Å × 80 Å × 80 Å water box. To allow sufficient sampling, the full range of NZ–OH distances from 2.5 Å to 6.0 Å was divided into eight windows with 0.5-Å intervals. At each window, a harmonic bias potential with a force constant of 20 kcal·mol^−1^·Å^−2^ was applied. Each simulation consisted of a 50-ps equilibration and 100-ps production time. Finally, the results from all windows were combined by using the weighted histogram analysis method [54]. A similar procedure was used to determine the strength of interaction between Arg206 and Glu45. However, two distances, NH1-OE1 and NH2-OE2, were used as the reaction coordinates for the two constrained backbone conformations, with one corresponding to the starting structure (the first homology model) and the other corresponding to the final conformer from the TMD simulation. Simulations were also performed on a truncated system: a single subunit from the full pentameric receptor with both ligand-binding and transmembrane domains embedded in a 50 Å × 50 Å POPC bilayer, then solvated with a 50 Å × 50 Å × 120 Å water box. To further reduce the computational cost, the NH1-OE1 distance was only varied between 2.5 Å and 3.5 Å, while the NH2–OE2 distance was allowed to sample the full range from 2.5 Å to 6.0 Å. We assumed that NH1 and OE1 always engaged in hydrogen bonding if only one hydrogen bond was possible (Figure 4A and 4B).

### Data analysis

**Figure 12:**
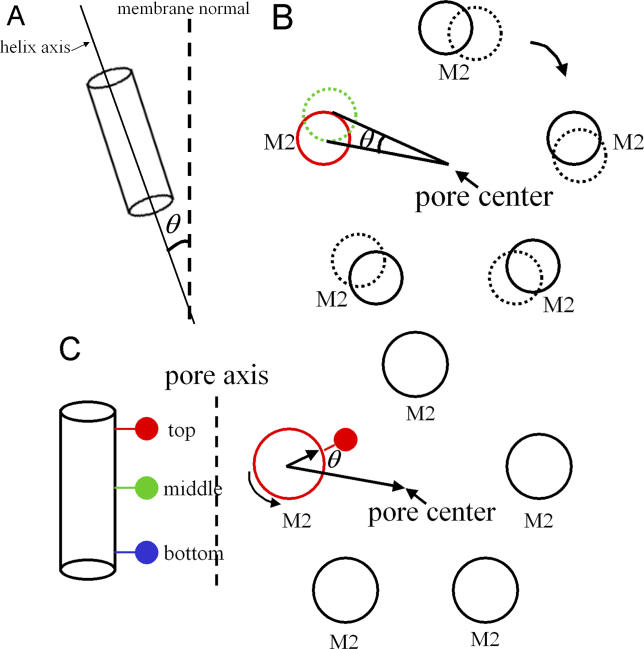
A Schematic Representation of Helix Tilting, Rotation around the Channel Axis, and Self-Rotation Angles (A) Helix tilting angle. (B) Rotation around the channel axis angle. (C) Self-rotation angle.

Pore radius profiles were determined with the program HOLE [55]. The tilt angle of M2 was defined as the angle between the principal axis of M2 and the membrane normal (Figure 12A). Prior to calculating helix rotational angles, the pore domain was aligned with the *z*-axis and the coordinates projected onto the *xy* plane. The rotation around the channel axis was measured as the angle formed by three centers of mass: the center of M2 for the current structure, the center of the pore, and the center of M2 for the reference structure (Figure 12B). The self-rotation of individual residues was defined as the angle formed by the C^α^ atom of the current structure, the center of M2, and the corresponding C^α^ atom in the reference structure (Figure 12C). All figures were prepared with the program VMD [56].

## Supporting Information

### Accession Numbers

Protein Data Bank (http://www.pdb.org) codes for the following genes are: AChBP (1UV6), AChBP structure with carbamylcholine bound (1UV6), Torpedo nAChR (2BG9), and *Torpedo* receptor structures (2BG9).

## Abbreviations

AChBP: acetylcholine binding protein
MD: molecular dynamics
nAChR: 7 nicotinic acetylcholine receptor
PMF: potential of mean force
TMD: targeted molecular dynamics
